# Noninvasive Proximal Adhesive Restoration in the Treatment of Non-cavitated Interproximal Incipient Carious Lesions: A Case Report

**DOI:** 10.7759/cureus.41542

**Published:** 2023-07-07

**Authors:** Waseem W Radwan

**Affiliations:** 1 Restorative Dentistry and Endodontics, Riyadh Elm University, Riyadh, SAU

**Keywords:** molar teeth., proximal caries, case-report, nipar, non-invasive proximal adhesive restoration

## Abstract

The noninvasive proximal adhesive restoration (NIPAR) technique is a noninvasive approach that utilizes a one-component universal adhesive for infiltration and a filled flowable resin for impermeable sealing. This technique offers several benefits. The noninvasive treatment approach is particularly significant in restorative dentistry, as it entails identifying and managing caries in their initial phases. The efficacy of the noninvasive proximal adhesive restoration technique is limited to non-cavitated lesions. In this case report, a 24-year-old male patient with no relevant medical conditions with a history of dental extractions and restorations visited the University of Geneva Dental School for a complete dental check-up. Orthopantomogram (OPG) and bitewing radiographs revealed multiple initial proximal caries in teeth # 37 (mesial), # 36 (mesial & distal), and # 35 (distal). In addition, DIAGNOcam (KaVo) was used as a diagnostic tool to establish proximal caries' progression. After discussing treatment options with the patient, a decision was made to treat the lesions at the enamel level using NIPAR.

## Introduction

Dental caries continues to be a highly prevalent disease among individuals of all ages, as evidenced by its impact on the elderly and the young [1]. Initial caries lesions are the earliest observable sign of enamel caries and are also referred to as white spot lesions. These lesions can manifest within two weeks following the accumulation of plaque [2]. There are both invasive and noninvasive treatment modalities for addressing early-stage carious lesions. Porcelain laminate veneers are a viable option for managing esthetic lesions that fail to respond satisfactorily to noninvasive interventions. This invasive treatment approach has been documented in the literature [3-4].

Nevertheless, these techniques are commonly linked with an overabundance of tissue loss. Conservative treatment options that aim to remineralize the initial caries lesions are considered noninvasive [5]. Several clinical studies have indicated that the remineralization processes do not result in any cosmetic enhancements, as assessed by the criteria of the International Caries Detection and Assessment System [6]. In addition to the techniques above, the resin infiltration method has demonstrated efficacy in various clinical and laboratory investigations and is categorized as a micro-invasive modality [7,8]. Resin infiltration is a concept that involves the utilization of resins with low viscosity to occupy the porous configuration of the primary enamel carious lesions that possess an unimpaired surface layer [9,10] in situations where the lesion's depth intensifies and results in cavity formation, the utilization of resin infiltration alone is inadequate. Composite resins must be employed to restore the cavity [11]. However, the noninvasive proximal adhesive restoration (NIPAR) technique, which combines infiltration and sealing, presents a viable treatment alternative for non-cavitated proximal lesions. This approach offers the benefits of infiltration and sealing without requiring invasive procedures [12]. Utilizing a variety of therapeutic approaches would be prudent for this specific scenario. The report demonstrates the benefits of integrating two distinct preventive approaches as a noninvasive proximal adhesive restoration. Hence this case report aims to demonstrate the application of a new noninvasive proximal adhesive restoration technique (NIPAR) in treating non-cavitated interproximal incipient caries lesions.

## Case presentation

A 24-year-old male patient visited the University of Geneva Dental School for a complete dental check-up. The medical history of the patient revealed no significant abnormalities. Patient habits revealed that he smokes one pack of cigarettes daily, brushes his teeth once daily, and never uses dental floss, despite having good oral hygiene. However, past dental history indicated the previous visit to a dentist for extraction and restoration. The oral examination showed extraction of wisdom teeth and restoration in #26. The plaque accumulation was minimal, with no gingival inflammation and periodontal pockets. Oral mucosa and tongue were within normal limits.

**Figure 1:**
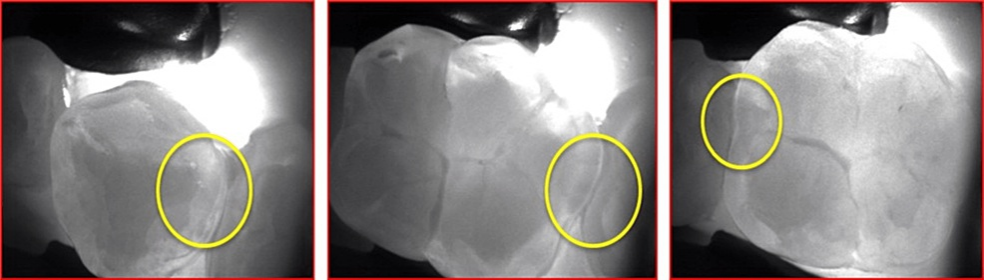
DIAGNOcam KaVo showing initial proximal caries in third quadrant

**Figure 2:**
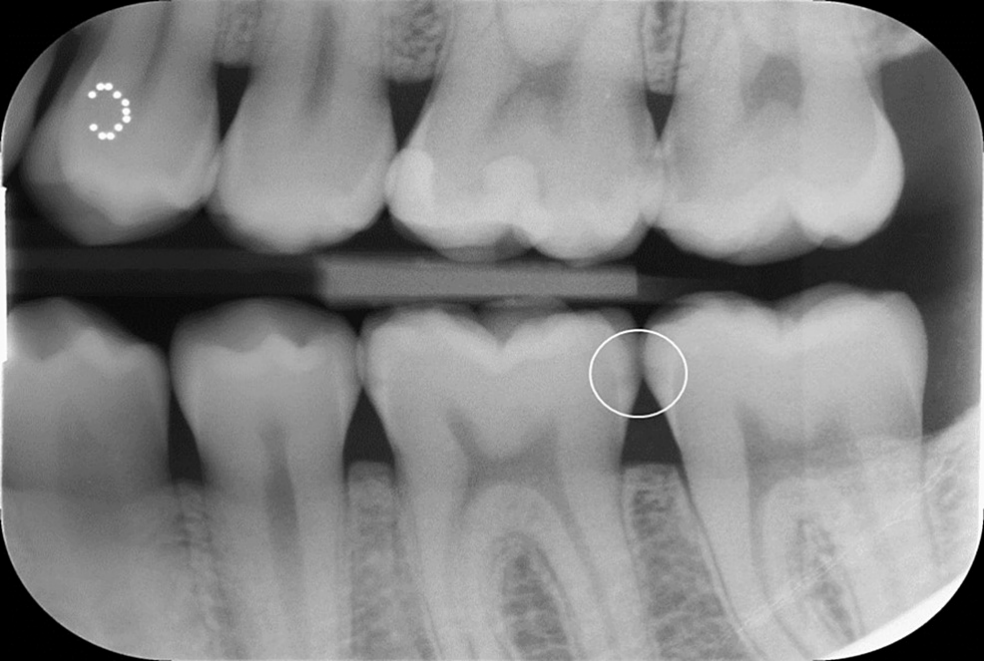
Bitewing radiograph showing initial proximal caries in teeth # 37 (MS) # 36 (MS and DS) and 35 (DS) #: tooth number, 37: Permanent left mandibular second molar,  36: permanent left mandibular first molar, 35: permanent left mandibular second premolar, DS: distal surface, MS: mesial surface

Orthopantomogram (OPG) and bitewing radiographs of the teeth were taken for the patient. The radiographs revealed multiple initial proximal caries, mainly in posterior teeth. In addition, DIAGNOcam (KaVo) was used as a diagnostic tool to establish proximal caries' progression. The bitewing radiographs showed initial proximal caries in teeth # 37 (mesial), # 36 (mesial & distal), and # 35 (distal) (Figures 1, 2

**Figure 3:**
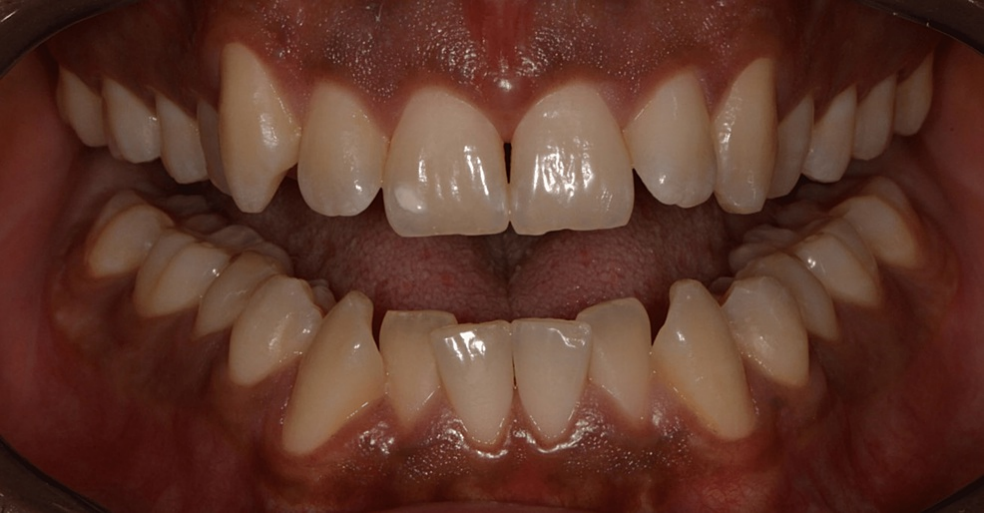
Mouth after oral prophylaxis

After reviewing treatment options with the patient, a decision was made to treat the lesions at the enamel level using NIPAR. Before beginning therapy, written informed consent was acquired. Oral prophylaxis was performed to remove food debris and dental plaque (Figure 3). In addition, oral hygiene instructions were given to the patient.

Procedure

**Figure 4:**
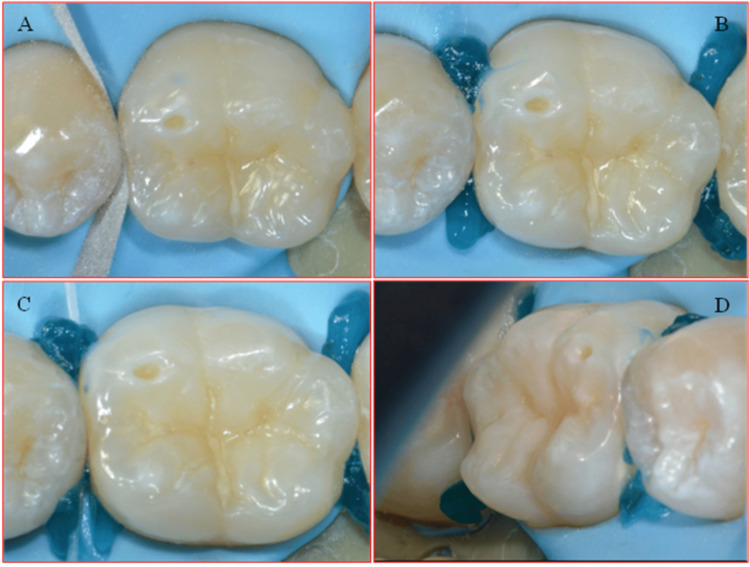
A. Diamond-coated metal finishing strip to prepare the proximal enamel surface. B. Application of 35% H3PO4 for 20 seconds. C. Complete penetration of H3PO4 using dental floss. D. Water Rinse for 20 seconds H3PO4: Phosphoric acid

A rubber dam (Nic Tone, Expert Tech Solution) was applied to isolate the teeth. A diamond-coated metal finishing strip was used to prepare the proximal enamel surface. The proximal enamel surface was etched by applying 35% Orthophosphoric acid (H3PO4) for 20 seconds. In order to ensure complete penetration of the acid, dental floss DF 834 (CURAPROX) was utilized, followed by a water rinse for 20 seconds (Figure 4).

**Figure 5:**
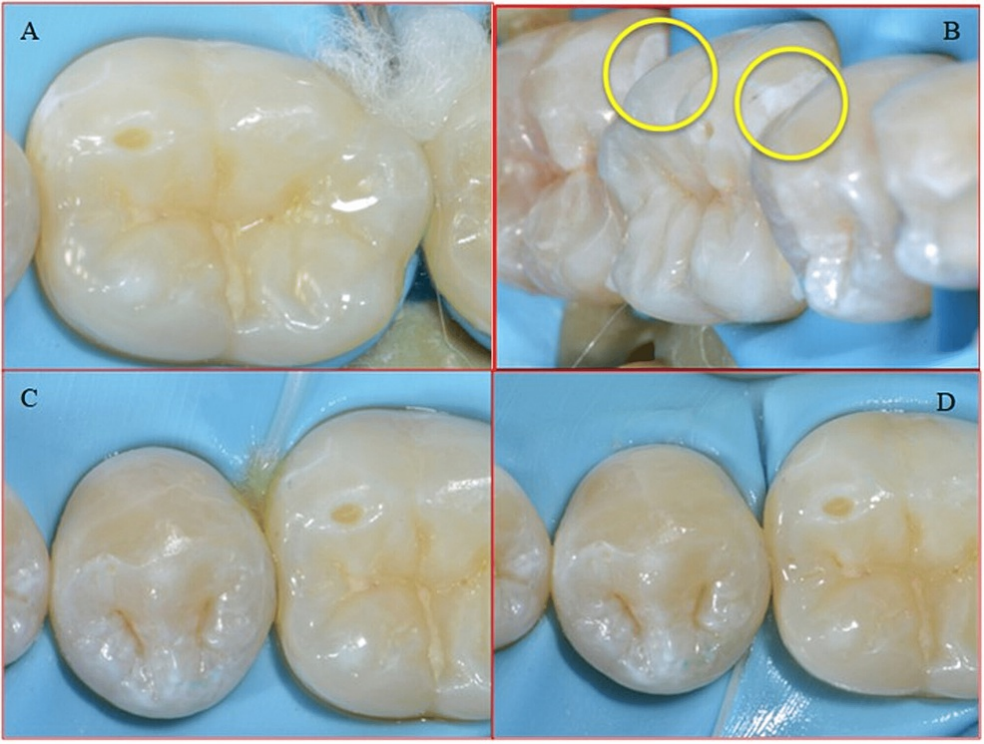
A. Ethanol for complete evaporation of water. B. White chalky appearance after acid etching. C. Application of bonding agent. D. Use of dental floss for complete adaptation of the bonding agent.

A small cotton pellet soaked in ethanol was placed for complete water evaporation. A white chalky appearance was seen as a result of the acid etch reaction. The bonding agent (3M™ Scotchbond™ Universal Adhesive) is applied to the acid-etched enamel surface. Dental floss was used to adapt the bonding agent completely (Figure 5).

**Figure 6:**
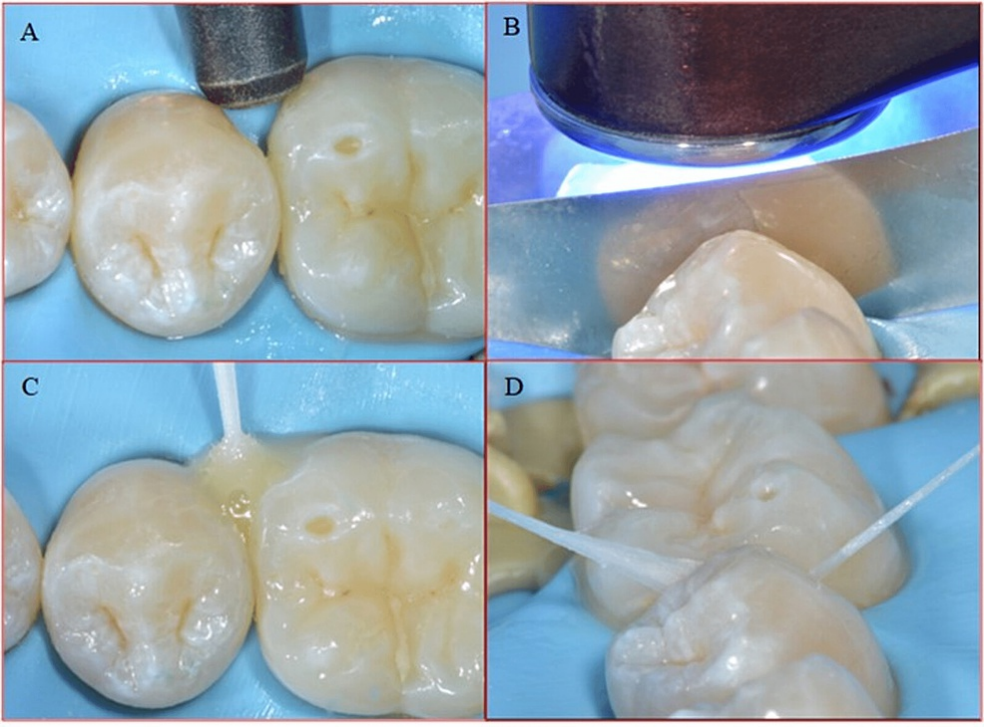
A. Air spray used to spread bonding agent in tight contacts. B. Metal matrix prevents adhesion of the proximal surface during polymerization. C. Flowable composite is applied as a restorative material. D. To enhance perfect penetration of the resin into proximal surface, dental floss is used.

The air was sprayed to spread the bonding agent uniformly, which was then light-cured for 30 seconds. The flowable resin (Tetric EvoFlow®, Ivoclar) was then applied as a restorative material on the enamel surface. In order to enhance the complete penetration of the resin into the proximal surface, dental floss was used, as shown in (Figure 6).

**Figure 7:**
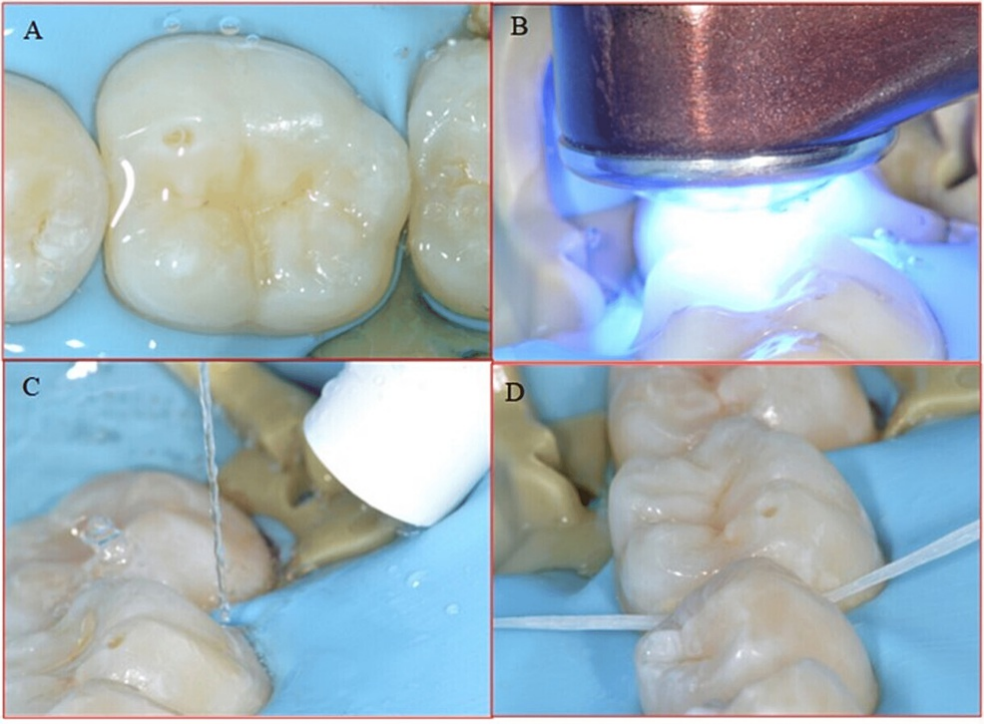
A. Glycerin gel is applied to inhibit oxidization. B. Polymerization for 20 seconds through the glycerin gel to avoid the oxygen inhibition layer. C. Washing excess glycerin. D. Use dental floss to remove excess resin.

Before initiating the polymerization reaction using light curing, a metal matrix (Polydentia, Switzerland) is used to prevent adhesion of the proximal surface during polymerization. The glycerine gel was applied to inhibit oxidation. The curing for 20 seconds was then carried out through the glycerine gel to avoid the oxygen inhibition layer. Excess glycerine was removed with water and dental floss (Figure 7).

**Figure 8:**
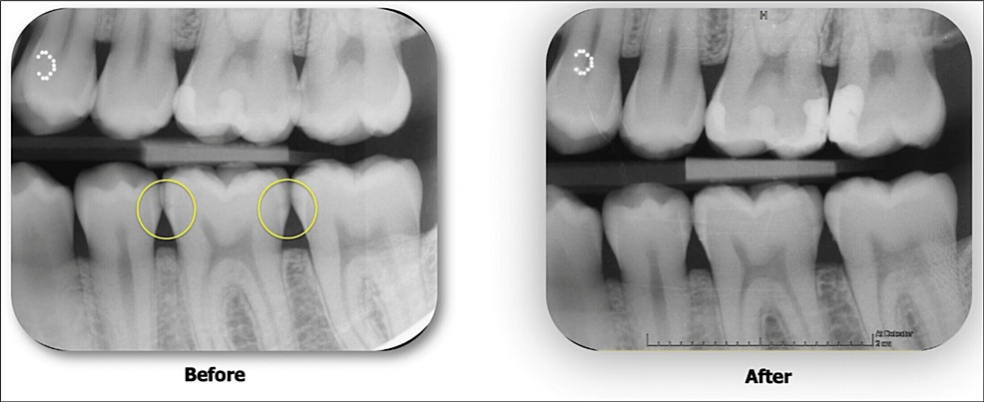
Bitewing radiographs showing before and after six months follow-up of treated incipient lesions

After six months, the patient was recalled for evaluation. The clinical examination revealed intact proximal restoration without any discoloration. While bitewing radiographs revealed the absence of progression of caries into surrounding tissues. The pre-treatment and six-month post-treatment radiographs of proximal incipient lesions are shown in Figure 8. Moreover, no complications were observed during or following the treatment procedure.

## Discussion

In this case report, the patient undergoing routine bitewing radiographic examination disclosed proximal non-cavitated carious lesions in teeth # 37 (mesial) # 36 (mesial & distal), and # 35 (distal), which were confirmed by DIAGNO cam. A non-invasive fluid resin treatment was initiated to restore these proximal lesions.

Using resin infiltration in combination with non-invasive interventions has been demonstrated to be a successful therapeutic strategy for managing non-cavitated proximal caries lesions in primary and permanent dentition. The accumulated evidence [13-15] was deemed to be solid and trustworthy with the incorporation of more recent assessments. Based on the reports, the treatment's long-term effectiveness was validated by monitoring lesion progression for most trials that evaluated lesions in permanent teeth over 36 months [16]. Additionally, one follow-up study conducted for seven years reported the effectiveness of fluid resin in treating non-cavitated proximal caries [13].

The noninvasive fluid resin infiltration procedure is a one-visit process that offers mechanical stabilization of demineralized enamel and exhibits enhanced resin penetration. The utilization of this approach aids in the prevention of lesion progression and reduces the likelihood of secondary caries. No instances of postoperative sensitivity or pulpal irritation, nor any cases of gingivitis or periodontitis, have been reported. The intervention mentioned above has been well-received by patients [15]. One of the reasons for this long success may be that the resin infiltration protects the initial lesions against acid attacks. According to various research studies, it has been suggested that resin-infiltrated enamel exhibits greater resistance toward acid attacks, consequently reducing the likelihood of caries lesion formation [17,18].

Using an abrasive strip on proximal surfaces, in conjunction with orthophosphoric acid etching, has been observed to improve the elimination of the hyper-mineralized layer. This technique also facilitates superior infiltration of adhesives into enamel lesions [12]. The utilization of unwaxed floss in the application of orthophosphoric acid in the proposed non-invasive proximal adhesive restoration protocol is recommended to facilitate optimal contact between the gel and the lesion surface. This approach effectively mitigates the formation of air pockets or bubbles on the lesion surface, which may impede the action of the gel [12]. The 35% phosphoric acid was chosen in this technique due to its high effectiveness in the removal of the hyper-mineralized layer after the use of an abrasive strip and thereby enabling better infiltration of adhesives into enamel lesions compared to the use of 15% Hydrochloric acid (HCl) for two minutes.

In the present case, Scotchbond Universal was applied, a single-component adhesive that the manufacturer reports to exhibit hydrophilic properties before polymerization, thereby facilitating optimal wetting of the tooth structure. After drying and curing, Scotchbond Universal demonstrates an exceptional conversion level and acquires a comparatively hydrophobic nature. According to the literature, a superficial filled resin coating offers superior protective properties compared to unfilled sealants. Utilizing a flowable resin to safeguard the infiltrated area is believed to augment further the level of protection against chemical and mechanical challenges that the lesion may face [18].

Based on prior observations [19,20], it has been noted that around 30-40% of lesions that penetrate the first third of dentin exhibit 43-45 micro cavitations. The non-invasive proximal adhesive restoration protocol can potentially mitigate the progression rate of dental caries by utilizing flowable composite material to fill these micro-cavities effectively.

The advantage of utilizing NIPAR is its ability to effectively demonstrate a greater extent of infiltration, which can be attributed to mechanical abrasion and the application of phosphoric acid. Additionally, NIPAR facilitates the achievement of an optimal surface seal through the utilization of flowable composite materials. Furthermore, NIPAR is a compelling therapeutic approach for managing non-cavitated or minimally cavitated lesions. However, a disadvantage of NIPAR is that it is technique sensitive and requires the use of a DIAGNOcam for proximal carious lesion identification. Moreover, the patient's good oral hygiene compliance is very much essential success of the treatment.

## Conclusions

The early intervention of the non-cavitated proximal caries is crucial to prevent the advancement of the lesion. The non-invasive proximal adhesive restoration (NIPAR) protocol demonstrated in this case report has the potential to offer a compelling therapeutic alternative for non-cavitated or even micro-cavitated proximal carious lesions. This case report outlines the steps involved in non-invasive proximal adhesive restorations of the proximal carious lesion.
